# Correction: Prevalence and Genetic Characterization of *Cryptosporidium* in Yaks in Qinghai Province of China

**DOI:** 10.1371/journal.pone.0097348

**Published:** 2014-05-16

**Authors:** The PLOS ONE Staff

## Notice of Republication

This article was republished on April 11, 2014, to correct formatting errors in genus and species names that were introduced during the typesetting process. These names should have been italicized throughout the article. Please download this article again to view the correct version. The originally published, uncorrected article and the republished, corrected article are provided here for reference.

## Supporting Information

File S1 Originally published, uncorrected article.
(PDF)

File S2 Republished corrected article.
(PDF)
