# Supplementary material for: Correction: Prevalence and Genetic Characterization of Cryptosporidium in Yaks in Qinghai Province of China
Source: PLoS One. 2014 May 16;9(5):e97348. doi: 10.1371/journal.pone.0097348 (PMC4024043; doi:10.1371/journal.pone.0097348)
Supplement: File S2 — Republished corrected article. (PDF) [file pone.0097348.s002.pdf]

PLOS ONE

Prevalence and Genetic Characterization of *Cryptosporidium* in Yaks in Qinghai Province of China

Rongsheng Mi[1], Xiaojuan Wang[1], Chunhua Li[2], Yan Huang[1], Peng Zhou[1], Zhengfeng Li[1], Mengtong Lei[2], Jinzhong Cai[2]*, Zhaoguo Chen[1]*

1 Key Laboratory of Animal Parasitology of Ministry of Agriculture, Shanghai Veterinary Research Institute, Chinese Academy of Agricultural Sciences, Shanghai, China,
2 Laboratory of Plateau Veterinary Parasitology, Veterinary Research Institute, Qinghai Academy of Animal and Veterinary Sciences, Xining, China

Abstract

The objective of this study was to determine the prevalence, species and subtypes of *Cryptosporidium* infecting yaks in the Qinghai Province of Northwestern China. The prevalence of *Cryptosporidium* spp. was detected by microscopy and nested-PCR. A total of 586 fecal samples were collected from yaks in 6 counties, of which 142 (24.2%) samples tested positive for *Cryptosporidium*. The small subunit (SSU) rRNA gene of fifty-five samples were amplified and sequenced successfully and demonstrated that *Cryptosporidium bovis* (31/55, 56.4%) was the most common species, followed by *C. parvum* (16/55, 29.1%) and *C. ryanae* (5/55, 9.0%). Mixed infections of *C. parvum* and *C. bovis* (n = 2), *C. ryanae* and *C. bovis* (n = 1) were also detected. All three species were found in yaks ranging in age from <1 year, 1–2 years, to >2 years. *Cryptosporidium* was most commonly detected in spring (28.4%), followed by summer (20.9%), then winter (17.5%). *Cryptosporidium parvum* positive samples were subtyped using the 60 kDa glycoprotein (gp60) gene. Subtypes IIaA15G2R1 (n = 8), IIaA16G2R1 (n = 2), IIaA14G1R1 (n = 1), IIaA14G2R1 (n = 1) and IIaA16G3R1 (n = 1) were detected. All of these subtypes are zoonotic, and may pose a potential threat to human health.

Citation: Mi R, Wang X, Li C, Huang Y, Zhou P, et al. (2013) Prevalence and Genetic Characterization of *Cryptosporidium* in Yaks in Qinghai Province of China. PLoS ONE 8(9): e74985. doi:10.1371/journal.pone.0074985

Editor: Dan Zilberstein, Technion-Israel Institute of Technology Haifa 32000 Israel., Israel

Received June 5, 2013; **Accepted** August 6, 2013; **Published** September 23, 2013

Copyright: © 2013 Mi et al. This is an open-access article distributed under the terms of the Creative Commons Attribution License, which permits unrestricted use, distribution, and reproduction in any medium, provided the original author and source are credited.

Funding: This study was supported in part by National S & T Major Program (Grant No. 2012ZX10004220-008), Shanghai Municipal Agriculture Commission (Grant No. 2005-3-4) and Basic Foundation for Scientific Research of State-level Public Welfare Institutes of China (Grant Nos. 2012JB16 and 2013JB13). The funders had no role in study design, data collection and analysis, decision to publish, or preparation of the manuscript.

Competing Interests: The authors have declared that no competing interests exist.

* E-mail: zhaoguochen@shvri.ac.cn (ZC); caijinz@vip.sina.com (JC)

Introduction

Cryptosporidiosis is a parasitic zoonosis which can cause sustained, serious and often life-threatening disease in immunosuppressed patients and animals [1]. *Cryptosporidium* spp. has been found to infect mammals, birds, reptiles, amphibians and fish [2]. *Cryptosporidium* infection occurs via many diverse transmission routes, such as direct contact with infected animals or ingestion of contaminated food and water [3]. Currently, nitazoxanide (NTZ) is approved for the treatment of cryptosporidiosis in children and immunocompetent adults in the United States, however, treatment failures have been reported and NTZ is ineffective for the treatment of immunocompromised individuals [4]. Halofuginone lactate (HL) is registered in several countries for the prevention of calf cryptosporidiosis, but the anti-*Cryptosporidium* activity and clinical benefit of HL are limited in the presence of other enteropathogens [5].

Most investigations have demonstrated that *Cryptosporidium parvum*, *C. bovis*, *C. ryanae* and *C. andersoni* are the major species in cattle [6], and the distribution of these species in cattle is age-related. *Cryptosporidium parvum* is mainly found in pre-weaned calves [7], *C. bovis* and *C. ryanae* in post-weaned calves [8], and *C. andersoni* is reported to primarily infect adult calves [9]. Other species such as *C. felis* [10], *C. scrofarum* [11], *Cryptosporidium* suis-like genotype [12], *C. suis* [12], *C. hominis* [13,14], *C. ubiquitum* [15] and *C. meleagridis* [16] were also described in cattle.

Yaks (*Bos grunniens*) reside at a higher altitude than any other member of the bovid family (2,500 to 6,000 m), and live in the Himalayan region (Nepalese Himalayas, Indian Kashmir, Mongolia, and the Qinghai-Tibetan plateau of China).The total global population of yaks is about 14 million, of which approximately 13 million (93%) domestic yaks live in China, and about 5 million yaks reside in the Qinghai Province in particular [17]. Yaks live in extremely poor conditions with average temperatures of −4°C to 8°C year-round, low oxygen content, high altitude and no completely frost-free periods. The products (milk, meat, bones, and wool) from yaks are a necessity for pastoral people. Therefore, research into the pathogens of yaks is important for the people of these regions.

In China, most studies of the prevalence of *Cryptosporidium* spp. in yaks were based on microscopical and serological detection [18–20], only two studies were published on *Cryptosporidium* species identification [21,22], but no studies with a large number of samples from yaks have been investigated, and no reports of subtype prevalence have been published. The aims of this study were to determine the prevalence and genotypes of *Cryptosporidium* spp. in yaks, and to investigate the regional differences, age-related and seasonal trends in *Cryptosporidium* infection of yaks in Qinghai Province, China.

Materials and Methods

Ethics Statement

This project was approved by the Shanghai Veterinary Research Institute Animal Ethics Committee. Before carrying out this work, we contacted the owners of all farms involved in the study and obtained their permission for collection of animal fecal samples. During specimen collection, all protocols used were consistent with the rules for Animal Care of the Chinese Academy of Agricultural Sciences.

Sample Collection

A total of 586 fecal specimens were collected from yaks between March 2008 and June 2012 in Dari (32°42′N, 98°15′E), Gangcha (37°19′N, 100°8′E), Gonghe (36°17′N, 100°37′E), Huangyuan (36°40′N, 101°15′E), Qilian (38°10′N, 100°15′E) and Zhiduo (33°51′N, 95°36′E) Counties in Qinghai Province, Northwestern China. The average altitude, annual rainfall, and average annual temperature of the 6 counties ranges from 3,200 to 4,500 m, 350 to 560 mm and −0.6 to 3.5°C, respectively. About 50 g fresh samples were collected immediately from the floor after animal defecation or directly taken from the rectum of the animals using sterile gloves, then put into a disposable plastic bag marked with farm name, serial number, host age and collection date. Samples were placed in ice boxes and transported to the laboratory, then stored in refrigerators at 4°C and processed as soon as possible. Most samples were collected from three age groups (<1 yr., 1–2 yr. and >2 yr. old yaks). These yaks had not previously been examined for *Cryptosporidium* infection and no history of cryptosporidiosis or administration of anti-cryptosporidial drugs was recorded.

Microscopy Detection

The samples from Gonghe and Huangyuan were examined via microscopy, as previously described by Chen et al. [23]. Briefly, 5 g of feces was diluted in distilled water and filtered through a strainer into a centrifuge tube. The filter was centrifuged at 1000×g for 5 min and the supernatant was discarded. The pellet was resuspended with 30 mL of Sheather's sucrose solution, and centrifuged at 400×g for 5 min. The supernatant was transferred to a microscopic slide by an iron loop and *Cryptosporidium* oocysts were detected with a light microscope under 400×magnification. Thirty-five positive samples from Gonghe and Huangyuan were selected randomly and preserved in 2.5% potassium dichromate and stored at 4°C.

DNA Extraction

All samples from Dari, Gangcha, Qilian, Zhiduo Counties, and 35 samples from Gonghe and Huangyuan Counties, were selected for genetic analysis. Genomic DNA was extracted from a 200 µL sample suspension by alkaline digestion and phenol-chloroform extraction [24]. The DNA was further purified using a QIAamp DNA Stool Mini kit (QIAGEN GmbH, Hilden, Germany) in accordance with the manufacturer's instructions, and stored at −20°C until further analysis.

PCR Amplification and Genotyping

Cryptosporidium species/genotypes were determined by nested PCR of the small subunit (SSU) rRNA gene and restriction fragment length polymorphism (RFLP) analysis, as described previously [25,26]. The secondary PCR products were digested with the restriction enzymes *Ssp* I, *Vsp* I and *Mbo* II (Fermentas Life Sciences, Lithuania, Vilnius, Lithuania) [21]. The digestion products were visualized by electrophoresis on 2% agarose gel. The species and genotypes were determined by comparing the banding patterns with published digest patterns [21]. The second amplification products were purified by AxyPrep DNA Gel Extraction Kit (Axygen Scientific, Hangzhou, China), then cloned into pMD 18-T Vector (TaKaRa Biotechnology, Dalian, China) and transformed into *Escherichia coli* DH5α. At least two positive clones from each sample were sequenced using ABI 3730×l DNA Analyzer (Applied Biosystems, Foster City, USA). Reference sequences of *Cryptosporidium* spp. were obtained from GenBank, and phylogenetic analysis was performed with MEGA 5.0 software (http://www.megasoftware.net/) using Distance analysis.

Subtype Identification

A fragment of the 60 kDa glycoprotein (gp60) gene was amplified by nested PCR as previously described [27]. The second PCR products were purified using AxyPrep DNA Gel Extraction Kit, cloned into pMD 18-T Vector and sequenced as per the SSU rRNA gene. The *Cryptosporidium* subtypes were identified as previously described by Sulaiman et al. [28].

Statistical Analysis

Statistical analysis of the prevalence of *Cryptosporidium* was carried out using IBM SPSS Statistics V21.0 for Windows (International Business Machines Corp, New York, USA). The differences between regions, ages and seasons were determined by Pearson's Chi-Square test (χ^2 test) analysis, and considered significant when $P<0.05$.

Results

Prevalence of *Cryptosporidium* Infection in Qinghai Province

Cryptosporidium was found in 142 of the 586 (24.2%) samples collected from six counties of Qinghai Province, 87 were diagnosed by microscopical examination, 47 by molecular analysis and eight by both methods (Table 1). The samples from Qilian, Gangcha, Dari and Zhiduo were diagnosed by PCR, and the samples from Gonghe and Huangyuan were diagnosed by microscopy. *Cryptosporidium* was detected in samples from all counties, with infection rates between 5.6% and 36.2%, with significant differences between different counties ($\chi^2 = 47.1$, $P<0.001$) (Table 1).

Prevalence of *Cryptosporidium* in Different Seasons

The prevalence of *Cryptosporidium* in different seasons was also investigated in the present study. The detection rate was highest in the spring (95/335, 28.4%), followed by summer (19/91, 20.9%) then winter (28/160, 17.5%), and varied significantly between seasons ($\chi2 = 7.61$, $P<0.05$) (Table 2).

Prevalence of *Cryptosporidium* Species

All samples that tested positive at the SSU locus were analyzed by restriction digestion, then sequenced. NCBI BLAST homology searches (http://blast.ncbi.nlm.nih.gov) identified *C. bovis* in 31 (56.4%) samples, *C. parvum* in 16 (29.1%) samples, *C. ryanae* in 5 (9.0%) samples, and mixed infections of either *C. parvum* and *C. bovis* or *C. ryanae* and *C. bovis* in 3 (5.5%) samples (Table 1).

Of the positive specimens, 22 sequences had 100% homology with the reference *C. bovis* sequence (GenBank accession No. AY741305), 15 with *C. parvum* (GenBank accession No. AF093493) and 4 with *C. ryanae* (GenBank accession No. EU410344). Other sequences shared 99% identity with the GenBank reference sequences, and all were submitted to the GenBank database under

Table 1. Prevalence and molecular characterization of *Cryptosporidium* spp. in different counties.

			Species					
County	No. Sample	No. Positive (%)	*C. parvum*	*C. bovis*	*C. ryanae*	*C. parvum*+ *C. bovis*	*C. ryanae*+ *C. bovis*	ND
Dari	71	4(5.6)	1	3	0	0	0	0
Gangcha	100	8(8.0)	1	5	2	0	0	0
Gonghe	50	10(20.0)	1	1	0	0	0	8
Huangyuan	235	85(36.2)	0	4	1	0	1	79
Qilian	71	20(28.2)	6	11	1	2	0	0
Zhiduo	59	15(25.4)	7	7	1	0	0	0
Total	586	142(24.2)	16	31	5	2	1	87

ND, not determined species.
doi:10.1371/journal.pone.0074985.t001

accession numbers KF128742 to KF128757. Phylogenetic analysis also supported the PCR-RFLP results and homology analysis.

Prevalence of *Cryptosporidium* Species in Different Host Age

The infection rates in yaks of different ages were studied. The highest prevalence of *Cryptosporidium* spp. was observed in yaks under the age of one year (24/88, 27.3%), followed by those between one and two years old (12/88, 13.6%), then those over two years of age (11/125, 8.8%), with significant differences between each age range ($\chi^2 = 34.9$, $P<0.001$).

Cryptosporidium bovis, *C. parvum* and *C. ryanae* were found in all age groups, but *C. bovis* was the most prevalent species in animals under two years of age, and *C. parvum* was the most prevalent species in animals over two years of age (Table 3). The genotyped samples from Huangyuan and Gonghe Counties were selected randomly from those samples in which *Cryptosporidium* was detected by microscopy, and the ages of the corresponding yaks was unknown.

Prevalence of *C. parvum* Subtypes

Thirteen of the 18 *C. parvum* positives were successfully amplified at the gp60 locus and were sequenced and aligned with GenBank reference sequences. We found that all sequences belonged to the *C. parvum* IIa subtype family. Altogether, five *C. parvum* IIa subtypes were found, IIaA15G2R1, IIaA16G2R1, IIaA14G1R1, IIaA14G2R1 and IIaA16G3R1, which were seen in 8, 2, 1, 1 and 1 animals, respectively. The unique sequences we acquired have been deposited in GenBank database under accession numbers KF128737 to KF128741.

Discussion

The rate of *Cryptosporidium* spp. infection in the yaks we studied was 24.2% (142/586), which is higher than the 10.4% infection rate reported by Zhou et al. [19], and lower than others ranging from 33.6% [20] to 39.7% [18] in Qinghai Province. *Cryptosporidium* was detected in samples from all six tested counties, and the prevalence varied significantly between the counties ($P<0.001$). The highest prevalence was found in Huangyuan (85/235, 36.2%), followed by Qilian (20/71, 28.2%), Zhiduo (15/59, 25.4%), Gonghe (10/50, 20.0%), Gangcha (8/100, 8.0%) and Dari (4/71, 5.6%). Previously, when serological techniques were used to detect *Cryptosporidium* infection rates of 30.4% (61/201) in Gonghe and 36.8% (82/223) in Qilian were found. We detected a lower prevalence with microscopic diagnosis (20.2% in Gonghe) or PCR (28.2% in Qilian) [20], in agreement with a previous report that serological tests have a higher prevalence rate than microscopic or PCR-based diagnosis [29]. The infection rates of 36.2% (85/235) found in Huangyuan was higher than that found in a previous study which utilized microscopic diagnoses and found an infection rate of 30.6% (26/85) [30]. This difference may be attributed to the different age of yaks in the population studied. The previous study sampled from only animals under the age of one year, but in our study the ages of animals were unknown in Huangyuan County. Given the similar climate of counties within the Qinghai Province, the inter-county variation in infection rates observed could be attributed to the age of the yaks sampled, the sampling season, feeding levels, pasture environment and the density of animals.

Most previous reports employed microscopical and serological diagnostic methods, and the species of *Cryptosporidium* circulating in yaks was not reported. In contrast we compared the genotypes of

Table 2. Prevalence and molecular characterization of *Cryptosporidium* spp. in different seasons.

			Species					
Season	No. Sample	No. Positive (%)	*C. parvum*	*C. bovis*	*C. ryanae*	*C. parvum*+ *C. bovis*	*C. ryanae*+ *C. bovis*	ND
Spring	335	95(28.4)	1	5	1	0	1	87
Summer	91	19(20.9)	4	12	3	0	0	0
Winter	160	28(17.5)	11	14	1	2	0	0
Total	586	142(24.2)	16	31	5	2	1	87

ND, not determined species.
doi:10.1371/journal.pone.0074985.t002

Table 3. Prevalence and molecular characterization of *Cryptosporidium* spp. in different host age groups.

			Species					
Age	No. Sample	No. Positive (%)	*C. parvum*	*C. bovis*	*C. ryanae*	*C. parvum*+ *C. bovis*	*C. ryanae*+ *C. bovis*	ND
<1 yr.	88	24(27.3)	5	15	2	2	0	0
1–2 yr.	88	12(13.6)	3	8	1	0	0	0
>2 yr.	125	11(8.8)	7	3	1	0	0	0
Unknown	285	95(33.3)	1	5	1	0	1	87
Total	586	142(24.2)	16	31	5	2	1	87

ND, not determined species.
doi:10.1371/journal.pone.0074985.t003

Cryptosporidium present in different regions, yak age groups and seasons. Fifty-five of the 142 samples that tested positive for *Cryptosporidium* were amplified and sequenced successfully, and three *Cryptosporidium* species were detected. *Cryptosporidium bovis* was the most common species (31/55, 56.4%), followed by *C. parvum* (16/55, 29.1%) and *C. ryanae* (5/55, 9.0%). Mixed infection of *C. parvum* and *C. bovis* (2/55, 3.6%), *C. ryanae* and *C. bovis* (1/55, 1.8%) were also identified, and *C. andersoni* was not detected in this survey. To our knowledge, this is the first identification of *C. parvum* and *C. ryanae* in yaks. *Cryptosporidium bovis* was previously found in an eight year old yak [21], and another study found a new *Cryptosporidium* genotype in a wild yak [22].

We found *C. bovis* to be the most prevalent species in the majority of counties (Dari, Gangcha, Huangyuan and Qilian), but in Gonghe and Zhiduo, *C. bovis* and *C. parvum* were equally prevalent. Most previous reports in pre-weaned calves found that *C. parvum* was the most common species [12,31–33], but in recent years *C. bovis* has been found to be the most prevalent species in pre-weaned calves in China [16,34], Nigeria [35], Japan [36,37], Sweden [38] and France [39]. We also found *C. bovis* to be most prevalent in yaks.

Similar to most studies of dairy calves [7,34,36,40,41], we found that *C. ryanae* was the least frequently detected species in yaks, in contrast to other studies of water buffaloes and beef calves in Egypt and Vietnam which reported that *C. ryanae* was most prevalent [42,43]. Moreover, a recent survey reported that *C. ryanae* was the only *Cryptosporidium* species identified in zebu cattle and water buffaloes in Nepal [44].

Previous surveys of adult calves found that *C. andersoni* was the predominant species in many countries including Japan, the United States and Canada [9,45,46], and this was also the case in China, according to three studies in Heilongjiang, Henan and Shaanxi Provinces [47–49]. In contrast we did not detect *C. andersoni* in yaks in this study. *Cryptosporidium andersoni* was also not found in adult calves in New Zealand, Denmark, the United States and Sweden [11,21,50,51].

In this study, we collected 285 samples from Huangyuan and Gonghe Counties in 2008, and *Cryptosporidium* was detected in 95 samples by microscopical examination. Thirty-five of these samples were selected randomly and preserved in 2.5% potassium dichromate, but only eight samples were amplified successfully by SSU rRNA gene in 2012. The low amplification success rate in this study can probably be attributed to the unsuitable preservation of samples. Further studies are therefore under way to collect larger numbers of fresh samples from yaks in these areas to genetically characterize *Cryptosporidium* spp. in Huangyuan and Gonghe.

The infection rate of *Cryptosporidium* spp. in yaks varied with age, consistent with previous surveys in dairy cattle which revealed that the prevalence of *Cryptosporidium* fell with increasing age [7,15,34,35]. We investigated the relationship between age and *Cryptosporidium* species infecting 47 of the 142 samples, and found that the species obtained in this study existed in all age groups. *Cryptosporidium bovis* was the most common species in yaks under two years of age, while *C. parvum* dominated in yaks over two years of age. A similar result has been observed in calves [11,50,52]. Previous studies reported that the prevalence of four common species (*C. parvum*, *C. bovis*, *C. ryanae*, and *C. andersoni*) in cattle varied with age. Eighty-five percent of pre-weaned calves were infected with *C. parvum*, 55% of post weaned calves were infected with *C. bovis* and 31% with *C. ryanae*, and 65% of heifers and mature cows were infected with *C. andersoni* in the United States [7–9]. However we did not observe such a trend in yaks, in agreement with the report in dairy cattle that *C. bovis* and *C. ryanae* are the major species found in pre- and post-weaned calves in the United States and China by Feng et al. [21].

Several studies have reported that seasonal shift could influence the *Cryptosporidium* infection levels in dairy cattle [34,41,53]. We found that the prevalence of *Cryptosporidium* spp. in yaks peaked in the spring (28.4%), declining in the summer (22.0%), and descended to the lowest levels in the winter (17.5%). This is in accordance with the report that *Cryptosporidium* infection of dairy calves peaks in spring [50], but is contrary to Szonyi et al. [41] and Wang et al. [34] who found that summer was the dominant infection season in the United States and China, while Hamnes et al. [53] found the highest prevalence in winter in Norway. The reasons for the seasonal variation in infection rates in yaks may be explained as follows: In spring in Qinghai Province, new grass has not yet grown, and yaks are fasted over the long winter. As a result these animals are generally malnourished and susceptible to infection [54]. At the same time, the weather in spring is beginning to warm and is more suitable for pathogen transmission, therefore spring has the highest incidence of disease over the year [54]. In the summer, food sources are plentiful and yak nutrition improves and with it their resistance to infection. In winter, the temperature can decline to $-20^{\circ}C$, preventing oocyst survival [55]. The saying that yaks are "alive in summer, strong in autumn, thin in winter, and tired in spring" [54] may be an accurate description of the seasonal variation in yak susceptibility to diseases. Unfortunately, we did not study the prevalence of *Cryptosporidium* in the autumn, so cannot comment on this season. We detected *C. bovis* infection in all studied seasons, in contrast to previous reports that *C. bovis* prevalence in pre-weaned calves peaked in autumn in China and in summer in the United States [34,41].

All 18 *C. parvum* positive specimens were subtyped by the GP60 gene, and 13 were amplified and sequenced successfully. Five subtypes (IIaA15G2R1 (n = 8), IIaA16G2R1 (n = 2), IIaA14G1R1 (n = 1), IIaA14G2R1 (n = 1) and IIaA16G3R1 (n = 1)) were found. This is the first report on the subtype distribution of *C. parvum* in yaks. The zoonotic subtype IIa has been found worldwide. Within the IIa subtype, IIaA15G2R1 was the most common subtype in calves which has been reported in many developed countries in North America [32,56] and Europe [12,15,31,40,57–60]. In contrast genetic characterization of *C. parvum* subtypes in dairy calves differed in China, where IIdA19G1 was the only subtype which had been identified previously [16,34]. We found that IIaA15G2R1 was the predominant subtype in yaks, as has previously been reported for calves in other Asian countries [37,61,62]. Other subtypes, IIaA16G2R1, IIaA14G1R1, IIaA14G2R1 and IIaA16G3R1, had been detected in previous studies of dairy calves. For example, IIaA16G2R1, IIaA14G2R1 and IIaA16G3R1 were found in the Netherlands [58], IIaA16G2R1 and IIaA16G3R1 in Canada, Spain and France [15,56,60], IIaA14G2R1 and IIaA16G3R1 in England [40] and IIaA16G2R1 and IIaA14G2R1 in Belgium [12]. However, subtype IIaA14G1R1 was mainly found in humans [57,63,64], and only one study has detected this subtype in calves (in Sweden [33]). All five subtypes identified in yaks in this study have been found in humans [64], suggesting that yaks may be involved in zoonotic transmission of *Cryptosporidium*.

In summary, we found that *Cryptosporidium* spp. was widespread in the yaks populating the Northwest of China. The overall infection rate was 24.2% for the Qinghai Province, and ranged from 8.0% to 36.2% in individual counties. Three species, *C. bovis*, *C. parvum* and *C. ryanae*, were found in every age group, and 56.4% of positive specimens contained *C. bovis*. To our knowledge, this is the first identification of *C. parvum* and *C. ryanae* in yaks. This is the first report on the subtype characteristics of *C. parvum* in yaks. We found that IIaA15G2R1 is the dominant subtype of *C. parvum* in yaks, which may pose a potential threat to human health.

Acknowledgments

We thank Wenkui Zhang and Yongjun Wang at Sanjiaocheng Sheep Breeding Farm of Qinghai Province and Ying Li at Qinghai University for their help in sample collecting.

Author Contributions

Conceived and designed the experiments: ZC JC. Performed the experiments: RM XW CL ZL. Analyzed the data: RM XW PZ YH ZC. Contributed reagents/materials/analysis tools: ZL CL JC ML. Wrote the paper: RM ZC.

References

1. Xiao L (2009) Overview of *Cryptosporidium* presentations at the 10th International Workshops on Opportunistic Protists. Eukaryot Cell 8: 429–436.
2. Xiao L, Fayer R (2008) Molecular characterisation of species and genotypes of *Cryptosporidium* and *Giardia* and assessment of zoonotic transmission. Int J Parasitol 38: 1239–1255.
3. Xiao L (2010) Molecular epidemiology of cryptosporidiosis: an update. Exp Parasitol 124: 80–89.
4. Rossignol JF (2010) *Cryptosporidium* and *Giardia*: treatment options and prospects for new drugs. Exp Parasitol 124: 45–53.
5. Almawly J, Prattley D, French NP, Lopez-Villalobos N, Hedgespeth B, et al. (2013) Utility of halofuginone lactate for the prevention of natural cryptosporidiosis of calves, in the presence of co-infection with rotavirus and *Salmonella* Typhimurium. Vet Parasitol 197: 59–67.
6. Fayer R, Santín M, Dargatz D (2010) Species of *Cryptosporidium* detected in weaned cattle on cow-calf operations in the United States. Vet Parasitol 170: 187–192.
7. Santín M, Trout JM, Xiao L, Zhou L, Greiner E, et al. (2004) Prevalence and age-related variation of *Cryptosporidium* species and genotypes in dairy calves. Vet Parasitol 122: 103–117.
8. Fayer R, Santín M, Trout JM, Greiner E (2006) Prevalence of species and genotypes of *Cryptosporidium* found in 1–2-year-old dairy cattle in the eastern United States. Vet Parasitol 135: 105–112.
9. Fayer R, Santin M, Trout JM (2007) Prevalence of *Cryptosporidium* species and genotypes in mature dairy cattle on farms in eastern United States compared with younger cattle from the same locations. Vet Parasitol 145: 260–266.
10. Bornay-Llinares FJ, da Silva AJ, Moura IN, Myjak P, Pietkiewicz H, et al. (1999) Identification of *Cryptosporidium felis* in a cow by morphologic and molecular methods. Appl Environ Microbiol 65: 1455–1458.
11. Langkjaer RB, Vigre H, Enemark HL, Maddox-Hyttel C (2007) Molecular and phylogenetic characterization of *Cryptosporidium* and *Giardia* from pigs and cattle in Denmark. Parasitology 134: 339–350.
12. Geurden T, Berkvens D, Martens C, Casaert S, Vercruysse J, et al. (2007) Molecular epidemiology with subtype analysis of *Cryptosporidium* in calves in Belgium. Parasitology 134: 1981–1987.
13. Smith HV, Nichols RA, Mallon M, Macleod A, Tait A, et al. (2005) Natural *Cryptosporidium hominis* infections in Scottish cattle. Vet Rec 156: 710–711.
14. Kang'ethe EK, Mulinge EK, Skilton RA, Njahira M, Monda JG, et al. (2012) *Cryptosporidium* species detected in calves and cattle in Dagoretti, Nairobi, Kenya. Trop Anim Health Prod 44 Suppl 1: S25–S31.
15. Follet J, Guyot K, Leruste H, Follet-Dumoulin A, Hammouma-Ghelboun O, et al. (2011) *Cryptosporidium* infection in a veal calf cohort in France: molecular characterization of species in a longitudinal study. Vet Res 42: 116.
16. Zhang W, Wang R, Yang F, Zhang L, Cao J, et al. (2013) Distribution and genetic characterizations of *Cryptosporidium* spp. in pre-weaned dairy calves in Northeastern China's Heilongjiang Province. PLoS One 8: e54857.
17. Sun WC, Luo YH, Ma HQ (2011) Preliminary study of metal in yak (*Bos grunniens*) milk from Qilian of the Qinghai Plateau. Bull Environ Contam Toxicol 86: 653–656.
18. Zhang J, Xu J, Shen X (2006) Investigation of *Cryptosporidium* infection in Cattle in Qinghai Province. Chin Qinghai J Anim Vet Sci 36: 16–17. (in Chinese).
19. Zhou C, He G, Zhang L (2009) Investigation on the *Cryptosporidium* infection in yak. Chin J Zoonoses 25: 389–390. (in Chinese).
20. Ma L, Lu Y, Cai Q, Wang G, Niu X, et al. (2011) Serological investigation on cryptosporidiosis of yak from Qinghai Province. J Domestic Anim Ecol 32: 47–49. (in Chinese).
21. Feng Y, Ortega Y, He G, Das P, Xu M, et al. (2007) Wide geographic distribution of *Cryptosporidium bovis* and the deer-like genotype in bovines. Vet Parasitol 144: 1–9.
22. Karanis P, Plutzer J, Halim NA, Igori K, Nagasawa H, et al. (2007) Molecular characterization of *Cryptosporidium* from animal sources in Qinghai province of China. Parasitol Res 101: 1575–1580.
23. Chen Z, Mi R, Yu H, Shi Y, Huang Y, et al. (2011) Prevalence of *Cryptosporidium* spp. in pigs in Shanghai, China. Vet Parasitol 181: 113–119.
24. Peng MM, Wilson ML, Holland RE, Meshnick SR, Lal AA, et al. (2003) Genetic diversity of *Cryptosporidium* spp. in cattle in Michigan: implications for understanding the transmission dynamics. Parasitol Res 90: 175–180.
25. Xiao L, Morgan UM, Limor J, Escalante A, Arrowood M, et al. (1999) Genetic diversity within *Cryptosporidium parvum* and related *Cryptosporidium* species. Appl Environ Microbiol 65: 3386–3391.
26. Xiao L, Bern C, Limor J, Sulaiman I, Roberts J, et al. (2001) Identification of 5 types of *Cryptosporidium* parasites in children in Lima, Peru. J Infect Dis 183: 492–497.
27. Alves M, Xiao L, Sulaiman I, Lal AA, Matos O, et al. (2003) Subgenotype analysis of *Cryptosporidium* isolates from humans, cattle, and zoo ruminants in Portugal. J Clin Microbiol 41: 2744–2747.
28. Sulaiman IM, Hira PR, Zhou L, Al-Ali FM, Al-Shelahi FA, et al. (2005) Unique endemicity of cryptosporidiosis in children in Kuwait. J Clin Microbiol 43: 2805–2809.
29. Nydam DV, Lindergard G, Guard CL, Schaaf SL, Wade SE, et al. (2002) Serological detection of exposure to *Cryptosporidium parvum* in cattle by ELISA and its evaluation in relation to coprological tests. Parasitol Res 88: 797–803.
30. Dou C (2007) Investigation on cryptosporidiosis of yak calves in Huangyuan County of Qinghai Province. Anim Husbandry Feed Sci: 14. (in Chinese).
31. Kváč M, Hromadová N, Květoňová D, Rost M, Sak B (2011) Molecular characterization of *Cryptosporidium* spp. in pre-weaned dairy calves in the Czech Republic: absence of *C. ryanae* and management-associated distribution of *C. andersoni*, *C. bovis* and *C. parvum* subtypes. Vet Parasitol 177: 378–382.
32. Xiao L, Zhou L, Santin M, Yang W, Fayer R (2007) Distribution of *Cryptosporidium parvum* subtypes in calves in eastern United States. Parasitol Res 100: 701–706.
33. Silverlås C, Bosaeus-Reineck H, Näslund K, Björkman C (2013) Is there a need for improved *Cryptosporidium* diagnostics in Swedish calves? Int J Parasitol 43: 155–161.
34. Wang R, Wang H, Sun Y, Zhang L, Jian F, et al. (2011) Characteristics of *Cryptosporidium* transmission in preweaned dairy cattle in Henan, China. J Clin Microbiol 49: 1077–1082.

35. Maikai BV, Umoh JU, Kwaga JK, Lawal IA, Maikai VA, et al. (2011) Molecular characterization of *Cryptosporidium* spp. in native breeds of cattle in Kaduna State, Nigeria. Vet Parasitol 178: 241–245.
36. Murakoshi F, Xiao L, Matsubara R, Sato R, Kato Y, et al. (2012) Molecular characterization of *Cryptosporidium* spp. in grazing beef cattle in Japan. Vet Parasitol 187: 123–128.
37. Murakoshi F, Tozawa Y, Inomata A, Horimoto T, Wada Y, et al. (2013) Molecular characterization of *Cryptosporidium* isolates from calves in Ishikari District, Hokkaido, Japan. J Vet Med Sci 75: 837–840.
38. Silverlås C, Blanco-Penedo I (2012) *Cryptosporidium* spp. in calves and cows from organic and conventional dairy herds. Epidemiol Infect: 1–11.
39. Rieux A, Chartier C, Pors I, Paraud C (2012) Dynamics of excretion and molecular characterization of *Cryptosporidium* isolates in pre-weaned French beef calves. Vet Parasitol 195: 169–172.
40. Brook EJ, Anthony HC, French NP, Christley RM (2009) Molecular epidemiology of *Cryptosporidium* subtypes in cattle in England. Vet J 179: 378–382.
41. Szonyi B, Bordonaro R, Wade SE, Mohammed HO (2010) Seasonal variation in the prevalence and molecular epidemiology of *Cryptosporidium* infection in dairy cattle in the New York City Watershed. Parasitol Res 107: 317–325.
42. Amer S, Zidan S, Feng Y, Adamu H, Li N, et al. (2013) Identity and public health potential of *Cryptosporidium* spp. in water buffalo calves in Egypt. Vet Parasitol 191: 123–127.
43. Nguyen ST, Fukuda Y, Tada C, Sato R, Duong B, et al. (2012) Molecular characterization of *Cryptosporidium* in native beef calves in central Vietnam. Parasitol Res 111: 1817–1820.
44. Feng Y, Karna SR, Dearen TK, Singh DK, Adhikari LN, et al. (2012) Common occurrence of a unique *Cryptosporidium ryanae* variant in zebu cattle and water buffaloes in the buffer zone of the Chitwan National Park, Nepal. Vet Parasitol 185: 309–314.
45. Budu-Amoako E, Greenwood SJ, Dixon BR, Barkema HW, McClure JT (2012) Occurrence of *Cryptosporidium* and *Giardia* on beef farms and water sources within the vicinity of the farms on Prince Edward Island, Canada. Vet Parasitol 184: 1–9.
46. Ikarashi M, Fukuda Y, Honma H, Kasai K, Kaneta Y, et al. (2013) First description of heterogeneity in 18S rRNA genes in the haploid genome of *Cryptosporidium andersoni* Kawatabi type. Vet Parasitol 196: 220–224.
47. Liu A, Wang R, Li Y, Zhang L, Shu J, et al. (2009) Prevalence and distribution of *Cryptosporidium* spp. in dairy cattle in Heilongjiang Province, China. Parasitol Res 105: 797–802.
48. Wang R, Ma G, Zhao J, Lu Q, Wang H, et al. (2011) *Cryptosporidium andersoni* is the predominant species in post-weaned and adult dairy cattle in China. Parasitol Int 60: 1–4.
49. Zhao GH, Ren WX, Gao M, Bian QQ, Hu B, et al. (2013) Genotyping *Cryptosporidium andersoni* in Cattle in Shaanxi Province, Northwestern China. PLoS One 8: e60112.
50. Learmonth JJ, Ionas G, Pita AB, Cowie RS (2003) Identification and genetic characterisation of *Giardia* and *Cryptosporidium* strains in humans and dairy cattle in the Waikato Region of New Zealand. Water Sci Technol 47: 21–26.
51. Silverlås C, Näslund K, Björkman C, Mattsson JG (2010) Molecular characterisation of *Cryptosporidium* isolates from Swedish dairy cattle in relation to age, diarrhoea and region. Vet Parasitol 169: 289–295.
52. Helmy YA, Krücken J, Nöckler K, von Samson-Himmelstjerna G, Zessin KH (2013) Molecular epidemiology of *Cryptosporidium* in livestock animals and humans in the Ismailia province of Egypt. Vet Parasitol 193: 15–24.
53. Hamnes IS, Gjerde B, Robertson L (2006) Prevalence of *Giardia* and *Cryptosporidium* in dairy calves in three areas of Norway. Vet Parasitol 140: 204–216.
54. Long RJ, Zhang DG, Wang X, Hu ZZ, Dong SK (1999) Effect of strategic feed supplementation on productive and reproductive performance in yak cows. Prev Vet Med 38: 195–206.
55. King BJ, Monis PT (2007) Critical processes affecting *Cryptosporidium* oocyst survival in the environment. Parasitology 134: 309–323.
56. Trotz-Williams LA, Martin DS, Gatei W, Cama V, Peregrine AS, et al. (2006) Genotype and subtype analyses of *Cryptosporidium* isolates from dairy calves and humans in Ontario. Parasitol Res 99: 346–352.
57. Soba B, Logar J (2008) Genetic classification of *Cryptosporidium* isolates from humans and calves in Slovenia. Parasitology 135: 1263–1270.
58. Wielinga PR, de Vries A, van der Goot TH, Mank T, Mars MH, et al. (2008) Molecular epidemiology of *Cryptosporidium* in humans and cattle in The Netherlands. Int J Parasitol 38: 809–817.
59. Imre K, Lobo LM, Matos O, Popescu C, Genchi C, et al. (2011) Molecular characterisation of *Cryptosporidium* isolates from pre-weaned calves in Romania: is there an actual risk of zoonotic infections? Vet Parasitol 181: 321–324.
60. Quilez J, Torres E, Chalmers RM, Robinson G, Del CE, et al. (2008) *Cryptosporidium* species and subtype analysis from dairy calves in Spain. Parasitology 135: 1613–1620.
61. Abe N, Matsubayashi M, Kimata I, Iseki M (2006) Subgenotype analysis of *Cryptosporidium parvum* isolates from humans and animals in Japan using the 60-kDa glycoprotein gene sequences. Parasitol Res 99: 303–305.
62. Nazemalhosseini-Mojarad E, Haghighi A, Taghipour N, Keshavarz A, Mohebi SR, et al. (2011) Subtype analysis of *Cryptosporidium parvum* and *Cryptosporidium hominis* isolates from humans and cattle in Iran. Vet Parasitol 179: 250–252.
63. Jex AR, Gasser RB (2008) Analysis of the genetic diversity within *Cryptosporidium hominis* and *Cryptosporidium parvum* from imported and autochtonous cases of human cryptosporidiosis by mutation scanning. Electrophoresis 29: 4119–4129.
64. Herges GR, Widmer G, Clark ME, Khan E, Giddings CW, et al. (2012) Evidence that *Cryptosporidium parvum* populations are panmictic and unstructured in the Upper Midwest of the United States. Appl Environ Microbiol 78: 8096–8101.
